# The post-2015 development agenda for diabetes in sub-Saharan Africa: challenges and future directions

**DOI:** 10.3402/gha.v8.27600

**Published:** 2015-05-19

**Authors:** Andre M. N. Renzaho

**Affiliations:** School of Social Sciences and Psychology, University of Western Sydney, Penrith, NSW, Australia

**Keywords:** post-2015 development agenda, diabetes, sub-Saharan Africa, non-communicable diseases

## Abstract

**Background:**

Diabetes is one of the non-communicable diseases (NCDs) which is rising significantly across sub-Saharan African (SSA) countries and posing a threat to the social, economic, and cultural fabric of the SSA population. The inclusion of NCDs into the post-2015 development agenda along with the global monitoring framework provides an opportunity to monitor progress of development programmes in developing countries. This paper examines challenges associated with dealing with diabetes within the development agenda in SSA and explores some policy options.

**Design:**

This conceptual review draws from a range of works published in Medline and the grey literature to advance the understanding of the post-2015 development agenda and how it relates to NCDs. The paper begins with the burden of diabetes in sub-Sahara Africa and then moves on to examine challenges associated with diabetes prevention, treatment, and management in Africa. It finishes by exploring policy implications.

**Results:**

With regards to development programmes on NCDs in the SSA sub-continent, several challenges exist: 1) poor documentation of risk factors, 2) demographic transitions (rapid urbanisation and ageing), 3) the complementary role of traditional healers, 4) tuberculosis and the treatment of the acquired immunodeficiency syndrome as risk factors for diabetes, 5) diabetes in complex emergencies, 6) diabetes as an international development priority and not a policy agenda for many SSA countries, and 7) poorly regulated food and beverage industry.

**Conclusion:**

For the post-2015 development agenda for NCDs to have an impact, sufficient investments will be needed to address legislative, technical, human, and fiscal resource constraints through advocacy, accountability, political leadership, and effective public–private partnership. Striking the right balance between competing demands and priorities, policies, and implementation strategies hold the key to an effective response to diabetes in SSA countries.

Sub-Saharan Africa has frequently been assaulted by wars, conflicts, malnutrition, and communicable diseases. Consequently, emergency responses, nutritional interventions, and prevention of communicable diseases have been the focus of development goals. However, non-communicable diseases (NCDs) have remained neglected in most sub-Saharan African (SSA) countries even though they account for a significant share of the burden of disease and have recently emerged as the silent killer (1, 2).

Obtaining objective data on NCDs in SSA countries remains a challenge and the most recent evidence comes from the 2010 Global Burden of Disease (GBD) study (3, 4). The GBD study estimated that NCDs account for over 60% of global mortality and contribute to 54% of global disability-adjusted life years lost each year, with 80% of deaths and 90% of early preventable deaths occurring in low- and middle-income countries (3, 4). It is worth noting that data from the GBD study have some serious flaws that limit the external validity of the findings. These flaws have been comprehensively discussed by Byass and colleagues (5). The main flaws identified by the authors include the fact that the findings are hard to replicate as the GBD study's description is insufficient and the data are not publicly available, the selective nature of data on deaths in the GBD-2010 database (e.g. high likelihood of including regions with complete data), and most importantly the GBD study reported modelled estimates and not measurements; hence, the reported estimates may or may not reflect reality (5).

Notwithstanding the paucity of objective data on NCDs in SSA countries, the few available data suggest that NCDs such as diabetes has increased from less than 1% between 1960 and 1980 (6) to 8–13% in the 1990s across SSA countries (7). A recent meta-analysis has found that diabetes in SSA countries has reached epidemic proportions. The overall pooled prevalence was estimated at 5.7% for diabetes, 4.5% for impaired fasting glycaemia, and 7.9% for impaired glucose tolerance (8). Diabetes prevalence in SSA countries varies by rurality, from 1% in rural Uganda to 12% in urban Kenya, and by ethnicity, from 8% in Zimbabwe to 18%, in SSA countries with advanced economies or with significant numbers of Indian sub-populations such as South Africa, Kenya, and Seychelles (9, 10).

The annual loss associated with NCDs including diabetes is estimated at US$500 billion or roughly 4% of the gross domestic product (GDP) and is estimated to surpass US$ 7 trillion over the period 2011–2025 for low- and middle-income countries (11). In 2000, the total economic cost of diabetes in SSA countries was estimated at US$67.03 billion or US$8,836 per diabetes patient (12), and in some countries it was one and a half times higher than the annual income per head and 50 times higher than the government health expenditure per person in some countries (13). In 2011, the annual total economic cost of diabetes in most SSA countries was significantly greater than the per-capita GDP expenditure on health (14). Diabetes-related complications cause the biggest burden to the SSA health systems. Available data suggest that cardiovascular disease (CVD) is the most common cause of death among diabetes patients while complications such as retinopathy, neuropathy, and nephropathy range from 7 to 63%, 27 to 66%, and 10 to 83%, respectively (9). These statistics indicate that SSA countries’ health systems will struggle to cope with the increasing demands of managing diabetes and its complications, unless the disease is recognised as a national health priority and early detection and treatment strategies are implemented. The aim of this paper was to make a conceptual review that could increase our understanding of challenges associated with dealing with diabetes within the development agenda in the SSA continent and act as a framework to explore some policy options. A conceptual review, rather than a systematic review, was the most appropriate for this paper because the emphasis was not on ranking individual articles based on their methodological approaches or to establish the relative importance of each challenge (15). That is, prior understanding and knowledge of the field allowed the review to be undertaken in a more integrated way. The review drew from a range of works published in Medline and the grey literature to advance the understanding of the post-2015 development agenda and how it relates to NCDs.

## Challenges associated with diabetes prevention, treatment, and management in Africa

### Challenge 1: Poor documentation of risk factors

Rising prevalence of diabetes in SSA countries is associated with a nutritional transition from a high-fibre traditional diet to an energy-dense Westernised diet leading to four key metabolic changes, namely unhealthy weight gain, raised fasting blood glucose levels, raised blood pressure, and hyperlipidaemia, all of which increase the risk of NCDs. These changes remain poorly addressed in most SSA countries probably due to the governments’ heavy investment in the prevention of communicable diseases, primarily driven by donors prioritising them to achieve various development objectives (e.g. Millennium Development Goals–MDGs).

Such biased investments are ironic given that NCDs are responsible for approximately two out of every three deaths each year with 80% of NCD-related deaths occurring in low- and middle-income countries, affecting one-third of those at an employable age (i.e. younger than 60 years) (16). It has been estimated that increased fasting glucose levels alone are responsible for 22% of deaths due to coronary heart disease and 16% of deaths due to stroke, while tobacco, physical inactivity, unhealthy diet, and harmful alcohol use account for nearly 80% of CVD, and diabetes alone is responsible for 3.5% of NCD deaths (17). Addressing these modifiable risk factors common to both diabetes and CVD results in substantial health gains, is cost-effective, and could prevent lost productivity due to premature death with long-term substantial economic returns (16).

**Fig. 1:**
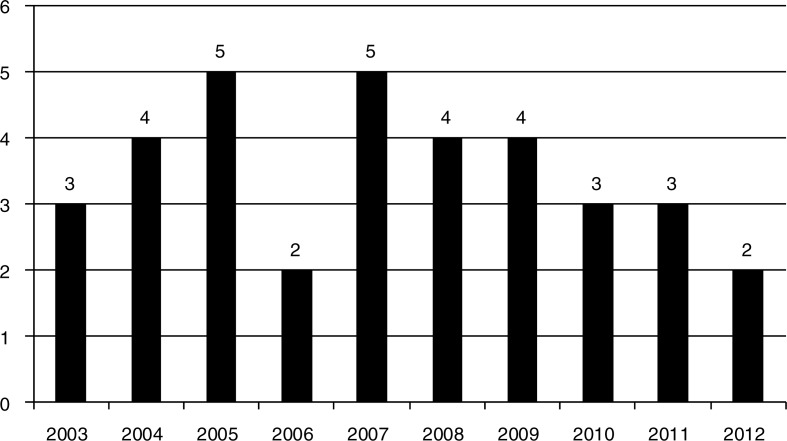
Number of STEPs surveys carried in SSA countries since 2003. Country-specific data were extracted from Ref. (20) and summarised for the purpose of this paper.

Objective data on these modifiable risk factors are missing. As Namusisi and colleagues (18) remarked: “*studies on the epidemiology of diabetes in sub-Saharan Africa are generally limited*” (p.2). Currently, there are no diabetes surveillance systems in SSA countries even though surveillance, through the collection, synthesis, and evaluation of data on diabetes risk factors, plays a significant role in informing prevention programmes, disease control, and treatment (18). For example, in its 2013 diabetes Atlas, the International Diabetes Federation considered 69 data sources from 29 SSA countries, out of which only 21 sources from 19 countries (39%) had usable data (19). In 2000, the World Health Organization (WHO) established the ‘stepwise’ approach (WHO STEPS) to chronic disease risk factor surveillance (STEPS). However, although the SSA region was the first WHO region to complete the STEPS training, uptake and adoption of the WHO STEPS has been slow in the region; and since 2000 only one country has obtained both baseline and follow-up data to depict trends (Fig. 1) (20).

### Challenge 2: Demographic transitions: rapid urbanisation and ageing

SSA countries have experienced the highest rate of urban growth in the developing world at 3.5% growth per year, from 14.7% in 1950 to 37% in 2010 and is projected to reach 60% by 2050 (21). Across SSA countries, increasing prevalence of diabetes has been associated with ageing and lifestyle changes which accompany urbanisation such as decrease in physical activity and changes in dietary patterns (6). Industrial processing resulting in highly refined, energy-dense, low fibre, and fatty foods populate the urban food markets replacing traditionally nutritious foods high in fibre, low in fat and salt. This could explain why the prevalence of diabetes in sub-Saharan Africa is significantly higher in the urban populations compared to their rural counterparts (6).

Therefore, as a consequence of increased urbanisation and an ageing population, the prevalence of diabetes will significantly increase across the sub-continent over time. Available data suggest that population ageing in SSA countries is already evident, as is the increasing prevalence of diabetes which peaks at 55 years or older (22). Diabetes-related inequities will arise due to co-morbidities associated with an ageing population, and the proliferation of slums and poverty which accompany urbanisation. With 60% of SSA populations projected to live in urban areas by 2050 (21), the long-term impacts of increased urbanisation and ageing on the health system have not been included in the SSA and global development agenda.

### Challenge 3: The complementary role of traditional healers

In SSA countries, two health systems coexist, the Western biomedical healthcare system and the traditional health care model (23). Available statistics suggest that traditional medicines constitute 80–90% of healthcare and interestingly, traditional healers have embraced biomedical knowledge and terminologies to maximise the effectiveness of their traditional treatment and healing practices (24). Meanwhile the biomedical health care system has been struggling to meet the challenges caused by diabetes, including inadequate diagnostic facilities, insufficient trained staff, and the high cost and limited availability of essential drugs such as insulin (25). Therefore, there has been a growing interest in the complementary roles of traditional healers within the biomedical health system in the management of diabetes especially given that traditional healers have played a prominent role in the treatment and management of human immunodeficiency virus/acquired immune deficiency syndrome (HIV/AIDS), tuberculosis (TB), and sexually transmitted diseases.

Some scholars have highlighted three advantages associated with acknowledging traditional healers as strategic partners in the fight against diabetes: they are highly accessible in poorly covered rural areas; they have high levels of community knowledge; there is preservation of the indigenous knowledge as well as cultural congruence (26, 27). Furthermore, their strategic role in the management of diabetes is emphasised by the fact that 80% of the SSA population including those with diabetes use traditional medicine as a source of primary care (24). In response, some researchers and policy makers have highlighted the dangers associated with incorporating traditional healers in the management and treatment of diabetes, while others have positioned traditional healers as potential assets to the health care system if they get adequate training and guidance. Some of the dangers identified in the literature include the risk of severe complications due to delayed diagnosis and treatment, deleterious practices involving the use of ineffective and toxic herbal remedies, and undermining the effectiveness of biomedical management of disease (28).

A systematic review evaluating the efficacy of herbal medicine for glucose control showed that although they were safe to use, there was inconclusive evidence to prove their efficacy (29). A pilot study evaluating the feasibility of integrating traditional healers into the biomedical health system in Cameroon found an improvement in their diabetes knowledge, practicing skills, and conducting community-based diabetes education after a 2-day training seminar. A total of 106 traditional healers attended the training workshops and a pre-test/post-test questionnaire was used to evaluate the intervention directly after the training and 8 months later on a sub-sample of 36 healers. Findings showed that there was an improvement in the traditional healers’ knowledge of diabetes and their ability to apply the lessons learnt such as referring patients for blood glucose tests, stopping the practice of scarifying patients with diabetes, and initiating education activities for patients, peers, and other people in their communities about diabetes prevention and treatment. The study found that traditional healers were not only enthusiastic about collaborating with the diabetes control programme but also asked for additional responsibilities. The authors concluded that “*healers could learn the prevention strategies of diabetes relatively rapidly and collaborate in health promotion* (30).”

### Challenge 4: AIDS treatment and TB as risk factors for diabetes

Another challenge associated with the management of diabetes in SSA countries is the relationship between NCDs and infectious diseases. Brown et al. (31) carried out a Multicentre Cohort Study that included 1,278 men (710 HIV-seronegative and 568 HIV-infected patients out of whom 411 were receiving highly active antiretroviral therapy (HAART)). They found that, during the 4-year observation period, after adjusting for age and body mass index, the incidence rate for diabetes among HIV-infected men on HAART was 4.7 cases per 100 person-years compared with only 1.4 cases per 100 person-years among HIV-seronegative men. They concluded that the observed 4-year risk of 10% was higher than previous estimates, hence supporting the urgent need to screen regularly for hyperglycaemia among people infected with HIV. The metabolic pathways that could explain the increased risk of diabetes among HIV-infected persons are multiple including the direct effects of HAART, the pro-inflammatory process of HIV, as well as the indirect effects of HAART such as changes in body fat distribution (32). While most studies have focused on diabetes as a risk factor for TB, emerging evidence suggests that the relationship is bidirectional, with a higher prevalence of diabetes found among TB-infected people (33). The increased risk of diabetes among TB-infected persons can be explained by multiple physiological pathways; however, the most documented ones are the increased risk of pancreatitis and insulin deficiency among TB-infected persons (34). Therefore, not only does TB increase the risk of diabetes but like many other infections, it also complicates diabetes management. For example, TB treatment regimens including Isoniazid have hyperglycaemic effects and interfere with insulin release, hence impairing glycaemic control among diabetics who are on this medication (32).

### Challenge 5: Diabetes in complex emergencies

To combat the effects of humanitarian crises caused by natural and man-made disasters, the international community has established 10 priorities (Box 1) to guide the planning and evaluation of the emergency response (35). However, these priorities do not include diabetes and other NCDs. Despite limited evidence on the management of diabetes in emergencies, Demaio and colleagues (36) argue that NCDs are associated with excess morbidity and mortality during complex emergencies due to several reasons: 1) people with NCDs are more vulnerable during emergencies (e.g. poor access to adequate food and appropriate disease management leading to quick deterioration), 2) disruption to routine health care services leading to acute complications, 3) post-emergency chronic comorbidity resulting from suboptimal management during emergencies, 4) and multifaceted impacts of NCDs and emergencies. They remark that the effect of NCDs in emergencies is life-long, noting: “*suboptimal management during and after a disaster not only has immediate health effects, but can also have lasting social and health ramifications. A lack of appropriate care for even a short period can result in greater levels of chronic morbidity and suffering, as well as poverty entrenchment*” (36, p. 4). Given that NCDs are known to be a barrier to economic development and equity, there is an urgent need to integrate diabetes and other NCDs into the existing emergency response-related policies and to formulate a transition plan to ensure the continuity of care post-emergency (36).

***Box 1*. Top priorities to address in emergencies (35).**

- Rapid assessment of the health status of the population
- Mass vaccination against measles
- Water supply and implementation of sanitary measures
- Food supply and implementation of specialised nutritional rehabilitation programmes
- Shelter, site planning, and non-food items
- Curative care based on the use of standardised therapeutic protocols, using essential drugs
- Control and prevention of communicable diseases and potential epidemics
- Surveillance and alert
- Assessment of human resources and training and supervision of community health workers
- Coordination of different operational partners

### Challenge 6: Diabetes a development priority and not a policy agenda for many SSA countries

Perhaps one of the biggest inadequacies in the development agenda is the omission of NCDs from the MDGs, which has occurred despite the documentation on NCDs-poverty vicious cycle and its link to all eight MDGs. In most developing countries, diabetes has been associated with increasing healthcare costs and loss of productivity (11, 37). However, the ability of most SSA countries to effectively respond to the diabetes epidemic has been hindered by limited funding, which again is a consequence of the omission of NCDs including diabetes from the development agenda. Recently, NCDs have gained considerable media coverage and been subject of political declarations, including the unanimous passing of the following declaration: ‘NCDs are one of the major challenges for development in the 21st century’, at both the 2011 UN high-level meeting on NCDs and the 2012 Rio+20 conference. The UN Task Team recognised that NCDs were a serious gap in the MDGs on the post-2015 development agenda and set them as a ‘priority for social development and investments in people’ (38), which led to the adoption of global NCD targets (i.e. reducing preventable deaths due to NCDs worldwide by 25% by 2025). There was also an acknowledgement that a global coordinating platform for NCDs needs to be established at the 65th World Health Assembly in 2012, spearheaded by the NCD Alliance (39). However, in the absence of national health priorities and commensurate funding, the ability of most SSA countries to effectively respond to the diabetes epidemic has been and will continue to be hindered by limited funding.

### Challenge 7: Regulating the food and beverage industry

The food and beverage industry is rarely mentioned when discussing NCDs in SSA countries. The rise in obesity in the continent has coincided with the increase in the consumption of fast foods and sugar-sweetened beverages (40). Since the turn of the century, SSA countries have grown as a viable market for most multinational fast food chains. Although data on the number of fast food stores across the continent are not available, it is estimated that the number of fast food restaurants has grown from none prior to 1980s to more than 1,000 Kentucky Fried Chicken outlets (41). In South Africa alone there are 65 fast food franchise brands with 5,400 outlets between them (42). In addition there are more than 900,000 retail shops selling Coca-Cola products and a staggering 78 million servings of Coca-Cola are consumed daily across the sub-continent (43). The increased consumption of fast foods and sugar-sweetened drinks is driven by the cultural desirability of animal products, including animal fats, sugar-sweetened foods and beverages, as well as intensive marketing activities, backed up by sales promotions. For example, SSA studies have found a strong *desirability of fried foods while stressing that boiled foods are uncivilized and unappetising* (44), p. 3). The emerging pattern is that of a decline in the local cultural food traditions, which are being replaced by multinational corporations importing industrialised countries’ food failures in the form of fast foods and sugar-sweetened beverages (45).

Such dietary transition, however, poses a serious challenge to addressing NCDs, especially diabetes in SSA countries. Cross-national data analysis from 75 countries showed that a 1% rise in soft drink consumption contributes to an additional 4.8% overweight, 2.3% obese, and 0.3% diabetic adults (46). In addition, De Vogli et al. (40) undertook a cross-national time series analysis from 25 countries examining the influence of market deregulation on fast food consumption and body mass index. They showed that a one-unit increase in per-capita annual fast food transactions was associated with an increase of 0.033 kg/m^2^ age-standardised body mass index while economic freedom independently predicted fast food consumption. They concluded that “*fast food consumption is an independent predictor of mean BMI in high-income countries. Market deregulation policies may contribute to the obesity epidemic by facilitating the spread of fast food*” (40, p. 99). Given such evidence, it is interesting to note that the post-2015 sustainable development goals are acknowledging the need to tackle NCDs without commensurate measures to reduce the threat posed by the multinational fast food and beverage chains. This is a particularly serious challenge for SSA countries where national policies regulating the food and beverage markets are either absent or inadequate.

## Policy implications and conclusion

In order for the post-2015 development agenda to effectively address NCDs, especially diabetes in Africa, a number of strategies, practice guidelines, NCD plans, and national policy agendas would need to be implemented. In terms of strategies, the focus would need to be on developing a robust standardised NCDs surveillance database to gather high-quality data necessary to inform the translation of research into policy and advocacy plans. The global monitoring framework to track progress in preventing and controlling major NCDs agreed upon by governments on the 7 November 2012 following the 2011 UN General Assembly's adoption of the political declaration on NCDs is a good start. The framework identified nine voluntary global targets as well as 25 indicators on diabetes and NCDs. Lessons learnt from the WHO STEPS indicate that implementing the global monitoring framework is not going to be straightforward.

A good surveillance system needs to be integrated into the national health information system and repeated over time to provide a trend and to measure exposure (e.g. risk factors and social determinants), outcomes (e.g. morbidity and mortality), and health system capacity and response (e.g. infrastructure and health plans and policies) (47). The WHO STEPS approach was developed to achieve some of these goals; however, several SSA countries in established baseline data in the early to mid-2000s but have not been able to repeat the survey to monitor trends over time. Similarly, many SSA countries have used findings from the WHO STEPS baseline surveys to develop NCD strategies and plans, but they remain non-operational, do not have measurable outcome targets, and rarely include evaluation components. Additionally, the components of health infrastructure to respond to NCDs remain unfunded or unimplemented. For example, surveys conducted by the WHO between 2000 and 2010 to assess the capacity of member states to prevent and control NCDs found that NCD funding stream was inexistent in 20 countries, lack of funding was prevalent, and these issues were, in particular, a serious problem in the African Region (47). Therefore, to avoid these challenges becoming evident in the global monitoring framework and a threat to its effectiveness, sufficient investments will be needed to address legislative, technical, human, and fiscal resource constraints through advocacy, accountability, political leadership, and effective public–private partnership. These measures would be a stepping stone to addressing the threat posed by the food and beverage industry, which remains poorly regulated in most SSA countries.

As the urban cities expand, the number of older people (60 years or older) will also increase significantly. Growing older brings with it long-term conditions, mainly NCDs, a critical burden to the healthcare system. Yet, SSA countries spend less on healthcare and are not well-equipped to meet the increased demand for healthcare that accompanies ageing. They also do not provide pensions, making older people a very socio-economically vulnerable sub-population in urban settings. Therefore, the negative impact of rapid urbanisation and ageing on the prevention, management, and treatment of diabetes in SSA countries, especially poor housing conditions, urban settlement overcrowding, and poor access to social and health services, will remain real if necessary policy responses are not put in place to deal with these challenges. Such policies will need to prioritise urban planning such as changing the urban built environment to facilitate active living, a supportive and enabling environment to meet the needs of older people, urban settlement planning and crowd control, and strategies to improve urban infrastructures and health services to maximise the effectiveness of diabetes health care services and diabetes management. While there are signs that progress has been made in introducing NCDs onto the development agenda post-2015, it would be important to have specific policy responses on NCDs in general and diabetes in particular for older people, especially those living in urban settings.

The role of traditional healers and traditional medicines in response to NCDs in general and diabetes in particular post-2015 cannot be discounted. Given the strong cultural importance attached to traditional healing across the sub-continent, the strong beliefs in witchcraft and supernatural forces in the genesis of diabetes, and the high social status and respect that traditional healers command in their communities, making traditional practices illegal as a policy option would be impossible and very hard to implement, and even more dangerous to patients. Diabetic patients who consult traditional healers and develop complications could be afraid to seek help within the biomedical model, for fear of getting the traditional healer arrested, even though many such complications are treatable by the biomedical model if diagnosed early. Another possible option could be a rigid registration process of traditional healers and a licensing system to regulate their practice which could be strengthened through training and ongoing education, accompanied by a manual which outlines harmful traditional practices and their health consequences, and guidelines on traditional healers’ role in ongoing patient education and care. The priority of such training should place a strong emphasis on traditional healers’ role as diabetes educators and in ensuring a strong and effective referral mechanism, especially when patients do not respond to traditional treatment or if there is any risk of developing complications.

There is a need to recognise that addressing diabetes effectively post-2015 may require that diabetes be also integrated into TB and AIDS management and treatment programmes and vice versa. Some of the lessons learnt over decades in relation to the treatment and management of TB and AIDS may be helpful in informing diabetes prevention, treatment, and management plans. This is particularly important given that the coexistence of communicable diseases and NCDs is going to characterise the SSA healthcare system for decades to come. Policies that promote integration, synergy, and coordination through a multispectral approach that incorporate diabetes into TB and AIDs treatment and management plans must be encouraged and prioritised.

Finally, the response to diabetes post-2015 needs to take into account the fate of diabetic patients in complex emergencies and in case of internal displacement due to civil conflicts or natural disasters. As indicated earlier, currently there are multitudes of guidelines and standards that govern the public health response in emergency situations, but these guidelines do not include NCDs, including diabetes. There is an urgent need to develop diabetes prevention guidelines and minimum standards to guide the treatment and management of diabetes in emergency situations. Given that public health interventions in emergency situations have as primary objective to save lives and prevent immediate mortality due to communicable diseases, an idea which is appealing to donors, the concept of diabetes treatment and management in emergency settings may be tough to sell to donors. However, one effective avenue could involve creating linkages between government-funded healthcare systems and the emergency response to maximise access to medication, to prevent disruption in the continuum of care. This may require reframing the debate to position diabetes as another priority in emergency situations and identifying political opportunities to mobilise resources.

In conclusion, the inclusion of NCDs into the post-2015 development agenda is a great step towards maximising of the effectiveness of development programmes in most developing countries. The global monitoring framework and its nine targets as well as 25 indicators provide an opportunity to monitor progress. However, there is no doubt that the coexistence of NCDs and communicable diseases will be a reality in SSA countries for the next 20 years or so, hence striking the right balance between competing demands and priorities, policies, and implementation strategies holds the key to effective response to diabetes in the sub-continent. Since diabetes is a threat to the social, economic, and cultural fabric of SSA populations and poses a serious burden to most SSA countries’ health systems, it needs to be integrated into national health priority areas. For the post-2015 development agenda to be effectively realised, concerted efforts are required to strengthen the capacity of the SSA health system, to build an effective political leadership, and to improve human capital.
